# Medical Gas Plasma Technology Combines with Antimelanoma Therapies and Promotes Immune‐Checkpoint Therapy Responses

**DOI:** 10.1002/advs.202303183

**Published:** 2023-08-04

**Authors:** Lea Miebach, Gabriella Melo‐Zainzinger, Eric Freund, Ramona Clemen, Alessandra Lourenco Cecchini, Sander Bekeschus

**Affiliations:** ^1^ Department of General, Thoracic, Vascular, and Visceral Surgery Greifswald University Medical Center 17475 Greifswald Germany; ^2^ ZIK *plasmatis* Leibniz Institute for Plasma Science and Technology (INP) 17489 Greifswald Germany; ^3^ Cancer Research Unit Boehringer Ingelheim Vienna 1121 Austria; ^4^ Department of Neurosurgery Wien University Medical Center Vienna 1090 Austria; ^5^ Laboratory of Molecular Pathology State University of Londrina Londrina 86051‐990 Brazil; ^6^ Clinic for Dermatology and Venerology Rostock University Medical Center 18057 Rostock Germany

**Keywords:** B16F10, CAP, immuno‐oncology, reactive oxygen species

## Abstract

Strategies to improve activity and selectivity are major goals in oncological drug development. Medical gas plasma therapy has been subject to intense research in dermatooncology recently. Based on partial gas ionization, this approach is exceptional in generating a variety of reactive oxygen species simultaneously that can be applied locally at the tumor side. It is hypothesized that combined gas plasma treatment can potentiate drug responses in the treatment of melanoma. Using a plasma jet approved as medical device in Europe, a systematic screening of 46 mitochondria‐targeted drugs identifies five agents synergizing in vitro and in vivo. Increased intratumoral leucocyte infiltration points to immunomodulatory aspects of the treatment, motivating to investigate responses to immune checkpoint blockade in combination with plasma. Tumor growth is monitored based on bioluminescent imaging, and single‐cell suspensions are retrieved from each tumor to characterize tumor‐infiltrating leucocytes using multicolor flow cytometry. Gene expression profiling is done using a validated NanoString panel targeting 770 genes specifically designed for immuno‐oncological research. Cell type abundancies are characterized from bulk RNA samples using the CIBERSORT computational framework. Collectively, the results indicate that local application of medical gas plasma technology synergizes with mitochondria‐targeted drugs and anti‐PD1 checkpoint therapy in treating melanoma.

## Graphical abstract

Medical gas plasma is a partially ionized gas operated at body temperature and generating various reactive species simultaneously. Using in vitro and in vivo melanoma models, gas plasma‐derived reactive species potently combine with novel mitochondria‐targeting drugs as well as antibody immune‐checkpoint therapy to increase tumor immunogenicity, antitumor immune responses, and tumor eradication.

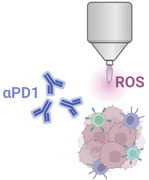

## 1 Introduction

Our scientific understanding of the pathogenesis of diseases evolves with technological and diagnostical advances, which, at the same time, drives the development of refined therapies interfering with deregulated signaling pathways tailored to the patient's situation. In oncology, the identification of cancer‐associated (and specific) microenvironmental and molecular signatures initiated a therapeutical shift from indiscriminate, cytotoxic agents to novel genome‐ and immune‐targeted therapies that improved outcomes of many malignancies in recent years. Yet, precision medicine is regularly challenged by on‐treatment tumor regrowth caused by secondary resistance mechanisms that limit therapeutic efficacies. In melanoma, a highly aggressive skin cancer often considered a model tumor in oncological research, therapeutic efficacy of standard‐of‐care BRAF (v‐raf murine sarcoma viral oncogene homolog B1)‐ and MEK (mitogen‐activated protein kinase kinase)‐inhibitors is often limited by extensively branched evolution of melanoma subclones.^[^ ^1^, ^2^ ^]^ Likewise, immune checkpoint blockade (ICB), the shining example of therapeutical advances in modern oncology, shows limited efficacy in 40% to 65% of patients, while further 43% of patients develop secondary resistance within three years.^[^ ^3^ ^]^ Considering tumor heterogeneity and the bottleneck effects of targeted therapies, growing evidence suggests that the precision medicine paradigm of cancer therapy requires the identification of eligible combination regimes rather than matching monotherapeutical approaches.^[^ ^4^, ^5^ ^]^

In the early 1990s, the unexpected finding of reactive oxygen species (ROS) limitation to promote cancer development,^[^ ^6^, ^7^ ^]^ followed by the discovery of ROS‐dependent cell death signaling elicited by an array of conventional chemotherapeutics and radiotherapy,^[^ ^8^, ^9^ ^]^ fostered the development of ROS‐generating therapy approaches such as photodynamic therapy (PDT)^[^ ^10^ ^]^ and medical gas plasma technology.^[^ ^11^ ^]^ Exploiting the concept of hormesis, such approaches aim at eliciting irreversible oxidative distress in cancer cells upon exposure to ROS at supraphysiological levels. While PDT is characterized by the local formation of singlet delta oxygen upon photoactivation of a systemically administered photosensitive agent, gas plasmas are exceptional in generating a multitude of highly reactive species simultaneously upon partial ionization of an operating gas. Plasma treatment of cancer cells has been subject of intense research in recent years showing antiproliferative, antimetabolic, senescence‐, and cell death‐elevating effects in diverse tumor models in vitro and in vivo.^[^ ^12^, ^13^, ^14^, ^15^, ^16^, ^17^ ^]^ Clinically, medical gas plasma therapy has been applied in the palliation of head and neck cancer patients in a series of clinical case reports conducted at the University Medical Center in Greifswald. Here, partial tumor remission (reduction in tumor size) could be observed in one‐third of patients, paralleled by an increase in patients’ life quality due to reduced odor, pain, and microbial load.^[^ ^18^, ^19^ ^]^ Despite direct tumor toxicity, gas plasma‐mediated tumor oxidation has been shown to create an inflammatory‐like environment able to trigger antitumor immune responses, e.g., through induction of immunogenic cell death (ICD) via ROS‐induced endoplasmatic reticulum (ER)‐stress^[^ ^20^, ^21^ ^]^ or formation of neoantigens via oxidation of biomolecules.^[^ ^22^, ^23^ ^]^

We hypothesized that local tumor oxidation using medical gas plasma technology could serve as a therapeutical sensitizer in oncological treatment regimens, improving response rates. Starting from a library of 46 mitochondria‐targeted drugs, five combination regimes were identified showing strong synergistic effects and subjected to further functional analysis in vitro and in vivo. In light of observed immune‐modulatory effects, medical gas plasma technology was combined with immune checkpoint blockade using pembrolizumab (anti‐PD1 antibody) in a syngeneic model of melanoma. Tumor growth was monitored based on bioluminescent imaging, and single‐cell suspensions were retrieved from each tumor to characterize tumor‐infiltrating leucocytes using multicolor flow cytometry. Gene expression profiling was done using NanoString technology and the PanCancer IO 360 Panel targeting 770 genes specifically designed for immuno‐oncological research. Cell type abundancies were characterized from bulk RNA samples using the CIBERSORT computational framework.

## 2 Results

### 2.1 Mitochondria‐Targeted Drugs Synergize with Medical Gas Plasma in the Treatment of Melanoma In Vitro and In Vivo

**Figure 1:**
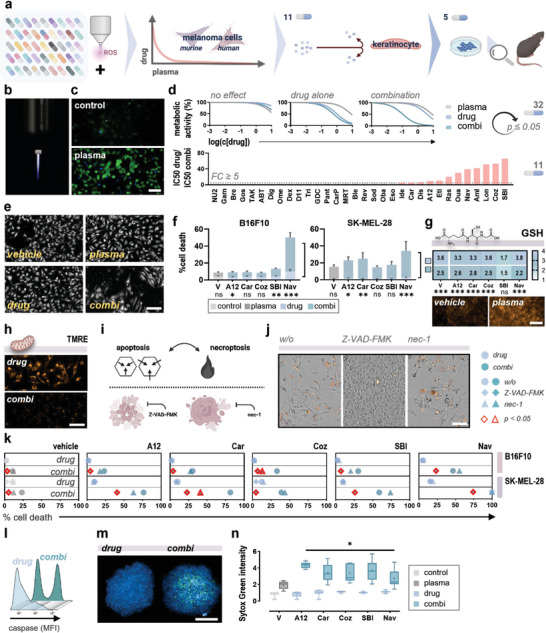
Mitochondria‐targeted drugs synergize with medical gas plasma treatment of melanoma cells in vitro. a) Study protocol. b) Plasma jet kINPen MED used in this study. c) Representative H_2_DCFDA and DAPI fluorescence images of control and gas plasma‐treated cells (scale bar = 50 µm). d) Representative dose–response curves and waterfall plot showing inhibitory concentration (IC) 50 ratios of monotreatment versus combination regimes in SK‐MEL‐28 human melanoma cells. Drugs were selected for significant combination effects (*p* < 0.05; *n* = 32) and IC50 fold change (FC) > 5 (*n* = 11). Graphs show mean. Statistical analysis was performed using one‐way analysis of variances (ANOVA). e) Representative digital phase contrast (DPC) images (scale bar = 100 µm). f) Cell death 24 h after treatment. Bar graphs show mean ± standard error of the mean (SEM). Statistical analysis was performed using two‐way ANOVA against vehicle (plasma) or drug monotreatments (combination regimes) (*p < 0.05, **p < 0.01, ***p < 0.001). g) Intracellular levels of reduced glutathione (GSH) 6 h after treatment (scale bar = 100 µm). Heat map shows mean. Statistical analysis was performed using two‐way ANOVA against vehicle (plasma) or drug monotreatments (combination regimes) (*p < 0.05, **p < 0.01, ***p < 0.001). h) Representative tetramethylrhodamine (TMRE) fluorescence images indicative of mitochondrial membrane potential (ΔΨm) 24 h after treatment. i) Cell death characterization. j) Representative brightfield and sytox orange fluorescence images (scale bar = 50 µm). k) Cell death 24 h after treatment. Graph shows mean. Statistical analysis was performed using two‐way ANOVA against vehicle (plasma) or drug monotreatments (combination regimes). l) Representative flow cytometry intensity histogram showing caspase 3 and 7 activity. m) Representative Hoechst and sytox green fluorescence images of tumor spheroids. n) 5–95 percentile box plots showing sytox green intensity in tumor spheroids 24 h after treatment. Mean is indicated as (+). Statistical analysis was performed using two‐way ANOVA against plasma monotreatment (*p < 0.05). ns = nonsignificant. ROS = reactive oxygen species. nec‐1 = necrostatin‐1. w/o = without. MFI = mean fluorescence intensity.

A library of 46 mitochondria‐targeted drugs was screened to identify promising candidates synergizing with medical gas plasma technology in the treatment of melanoma. A three‐step screening procedure was employed to identify substances with maximal combinational efficacy in murine and human melanoma cells while exhibiting low toxicity on non‐neoplastic human keratinocytes. Selected drug combination regimes were further investigated in vitro and in a syngeneic model of melanoma in vivo (**Figure** **1**a). Placed in the heart of applied redox biology, medical gas plasma technology is exceptional in generating a variety of ROS and RNS simultaneously (Figure 1b), eliciting oxidative stress in cells (Figure 1c) followed by induction of irreversible cell death signaling when applied at supraphysiological levels. Increased basal intracellular ROS levels occurring upon malignant transformation suggest a lowered effective threshold in cancer cells, entailing a potential and often described selectivity of medical gas plasmas and a high therapeutic index (Figure S1, Supporting Information). Based on the cellular metabolic activity 24 h after treatment, fourfold dose–response curves were generated for murine B16F10 and human SK‐MEL‐28 melanoma cells to identify substances without therapeutic efficacy (no effect), without synergistic effects (drug alone), and significant combination effects following exposure to gas plasma‐derived ROS (combination). Drugs synergizing with medical gas plasmas were chosen for further analysis (*n* = 32 drugs). Inhibitory concentration (IC) 50 ratios were calculated to identify drugs showing a leastwise fivefold decreased IC50 in combination versus monotreatments in human SK‐MEL‐28 (Figure 1d) and murine B16F10 melanoma cells (Figure S2a, Supporting Information), contributing to a set of 11 drug candidates. Combination regimes with a narrow therapeutic index were further excluded based on calculated IC50 ratios of combination treatments in non‐neoplastic HaCaT keratinocytes versus SK‐MEL‐28 melanoma cells (Figure S2b, Supporting Information). Thus, five compounds, A‐1210477 (A12), Carvedilol (Car), Cozymasei (Coz), SBI‐0206965 (Coz), and Navitoclax (Nav), were finally identified as promising drug candidates and further characterized in vitro and in vivo.

Combined drug–plasma therapy reduced cancer cell proliferation (Figure 1e) and enhanced tumor toxicity 6 h (Figure S2c, Supporting Information) and 24 h (Figure 1f) after treatment compared to drug monotreatments. Oxidative distress is characterized by an overwhelming consumption of cellular redox capacities, observed for the antioxidant tripeptide glutathione (GSH) predominantly after exposure to gas plasma‐derived ROS in our study (Figure 1g). Loss in mitochondrial membrane potential (ΔΨm) as a surrogate marker for mitochondrial dysfunction and irreversible oxidative damage was evaluated using tetramethylrhodamine (TMRE; Figure 1h). TMRE is readily sequestered by active mitochondria due to the dye's positive charge and indicated significant mitochondrial damage upon combinational treatment (Figure S1d, Supporting Information). Mitochondria are implicated in an expanding array of cellular processes and considered multifaceted regulators of cell death. Cell death modalities predominantly induced in combination regimes were further evaluated using the pan‐caspase, hence apoptosis, inhibitor Z‐VAD‐FMK, and the RIP‐1 kinase inhibitor necrostatin‐1 (nec‐1) for inhibition of necroptosis (Figure 1i). Algorithm‐based image analysis for segmentation of sytox orange^+^ melanoma cells (Figure 1j) revealed significant cell death inhibition in the presence of Z‐VAD‐FMK indicating apoptosis as the preferred mode of execution upon combined drug–plasma therapy (Figure 1k). This was underlined by increased activation of effector caspases 3 and 7 in combination regimes (Figure 1l). A superior efficacy was further confirmed in 3D melanoma spheroids (Figure 1m) outlining significant synergistic effects (Figure 1n).

**Figure 2:**
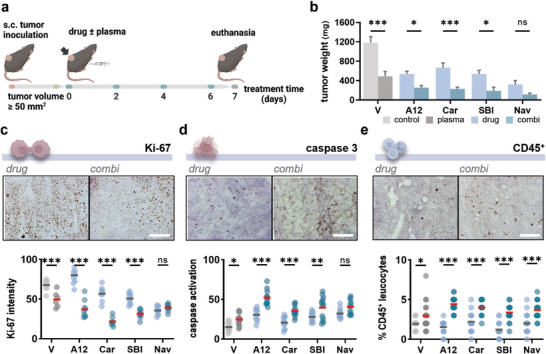
Mitochondria‐targeted drugs synergize with medical gas plasma treatment in a syngeneic melanoma model in vivo. a) Study protocol. b) Tumor weight. Bar graphs show mean ± standard error of the mean (SEM). Statistical analysis was performed using two‐way analysis of variance (ANOVA) against vehicle (plasma) or drug monotreatments (combination regimes), respectively (*p < 0.05, ***p < 0.001). c–e) Representative immunohistochemistry images of Ki‐67 (c), cleaved caspase 3 (d), and CD45 (e) staining and quantification thereof (scale bar = 200 µm). Graphs show mean and individual values. Statistical analysis was performed using two‐way ANOVA against vehicle (plasma) or drug monotreatments (combination regimes), respectively (*p < 0.05, **p < 0.01, ***p < 0.001). ns = nonsignificant.

Promising drug candidates were next investigated in a syngeneic model of melanoma in vivo. Tumor growth was induced by subcutaneous injection of 1 × 10^6^ B16F10 melanoma cells in the left flank of C57/Bl6 mice. Animals were randomly divided into ten groups after reaching a tumor volume of ≈50 mm^2^. Melanoma‐bearing mice received either (a) intraperitoneal drug monotreatment, (b) local gas plasma treatment, or (c) combination of both. Treatments were performed every other day in four treatment cycles until animals were sacrificed on day 7 (**Figure** **2**a). Strikingly, combined tumor toxic effects were observed in all treatment groups (Figure 2b). Histopathological analysis of cryosectioned tumor nodes further revealed a reduced proliferative index (Figure 2c) and increased activation of effector caspase 3 in line with previous experiments (Figure 2d). Moreover, combinational treatment increased the number of tumor‐infiltrating CD45^+^ leucocytes in all groups indicating immune modulatory aspects of the treatment (Figure 2e).

### 2.2 Gas Plasma Treatment Reduces Myeloid and Lymphoid Checkpoint Expression Levels on Melanoma Cells In Vitro and In Vivo

**Figure 3:**
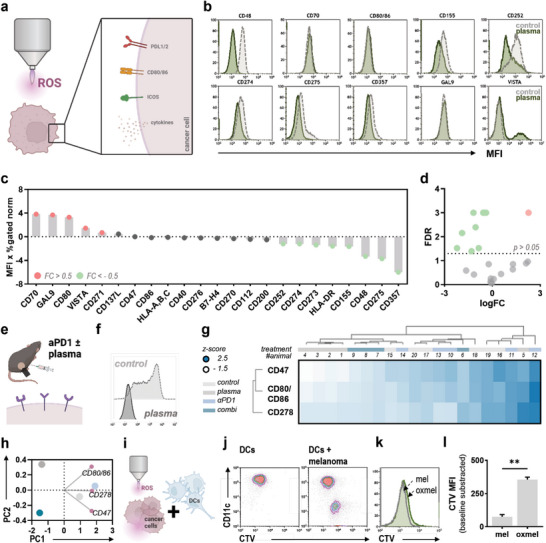
Gas plasma treatment reduces myeloid and lymphoid checkpoint expression levels on melanoma cells in vitro and in vivo. a) Study protocol. b) Representative flow cytometry intensity histograms showing tumoral expression of immunosuppressive surface markers. c) Waterfall plot showing fold change (FC) expression levels of immunosuppressive surface markers on human melanoma cells after gas plasma treatment. Bar graphs show mean. d) Volcano plot showing differentially (*p* > 0.05) up‐ (red) or downregulated (green) markers. Statistical analysis was performed using one‐way analysis of variances (ANOVA). e) Study protocol. f) Representative flow cytometry intensity histogram showing CD80/CD86 expression B16F10 melanoma tumors. g) WPGMA‐weighted hierarchical clustering of z‐scored surface expression of CD47, CD80/CD86, and CD278 on B16F10 melanoma tumors of individual animals after treatment with gas plasma, anti‐PD1 or the combination of both. h) Principal component analysis (PCA) calculated from z‐scored mean surface expression levels of CD47, CD80/CD86, and CD278 on B16F10 melanoma tumors of individual animals after treatment with gas plasma, anti‐PD1 or the combination of both. i) Study protocol. j) Representative flow cytometry cell trace violet (CTV) versus CD11c contour plots of CD11c^+^ dendritic cells (DCs) challenged with CTV‐labeled melanoma cells. k) Representative flow cytometry intensity histograms showing CTV fluorescence in CD11c^+^ DCs challenged with CTV‐labeled heat‐killed (mel) or oxidized melanoma cells (oxmel). l) Basline‐corrected CTV mean fluorescence intensity (MFI) in CD11c^+^ DCs challenged with heat‐killed (mel) or oxidized melanoma cells (oxmel). Bar graphs show mean ± standard error of the mean (SEM). Statistical analysis was performed using *t*‐test (**p < 0.01). norm = normalized. FDR = false discovery rate. PC = principal component. ROS = reactive oxygen species. αPD1 = anti‐PD1.

The previous findings highlighted the potential of combined drug–plasma therapy in the treatment of melanoma and indicated immune‐modulatory aspects of the treatment in vivo. Despite direct tumor‐toxic effects outlined above, gas plasma‐derived ROS have likewise been implicated to act as bona fide inducers of ICD and may facilitate formation of neoantigens via oxidation of biomolecules. In this light, we hypothesized that gas plasma treatment could potentiate responses to ICB in the treatment of melanoma. Following successful cross‐presentation and T cell priming, an inhospitable tumor microenvironment (TME) may preclude proper function of the expanded T cell repertoire, limiting the efficacy of ICB. This might include the aberrant expression of alternate immune checkpoints or coinhibitory receptors, immunosuppressive cytokines, and immune‐inhibitory metabolites. As the ICD‐inducing activity of gas plasmas is well described in the literature, we aimed to characterize the immune checkpoint expression profile in response to plasma‐mediated tumor oxidation (**Figure** **3**a). Flow cytometric screening (Figure 3b) of an array of 23 surface‐expressed checkpoint molecules (Figure 3c) revealed a significant downregulation for eight markers upon plasma treatment, while a significant upregulation was only found for CD70 (Figure 3d). Checkpoint expression signatures were further evaluated after gas plasma monotreatment and combined gas plasma–ICB in vivo (Figure 3e). Single cell suspensions were retrieved from excised B16F10 tumors and stained with monoclonal antibodies targeting CD47, CD80/CD86, and CD278 followed by flow cytometric analysis (Figure 3f). WPGMA‐weighted hierarchical clustering of checkpoint marker expression signatures on individual tumors revealed lower expression levels in groups receiving plasma monotreatment or the combination with anti‐PD1 ICB (Figure 3g), which was underlined by notable differences in the principal component (PC) 1 (Figure 3h). Propagation of an effector T cell response in draining lymph nodes requires successful antigen‐processing and presentation by antigen‐presenting cells (APCs). As outlined above, medical gas plasmas are considered bona fide ICD inducers, indicated by expression and release of damage‐associated molecular patterns (DAMPs) after ROS‐induced cell death that may attract APCs to the tumor side. In order to investigate cancer–immune cell interactions after plasma treatment, monocyte‐derived CD11c^+^ dendritic cells were challenged with cell trace violet (CTV)‐labeled plasma‐oxidized melanoma cells (oxmel; Figure 3i). Flow cytometric evaluation (Figure 3j) of CTV fluorescence in CD11c^+^ dendritic cells (Figure 3k), indicative of tumor uptake, indicated increased phagocytosis of oxmel versus heat‐inactivated melanoma cells (mel; Figure 3l).

### 2.3 Medical Gas Plasma Technology Synergizes with Anti‐PD1 Immune Checkpoint Blockade in a Syngeneic Model of Melanoma In Vivo

**Figure 4:**
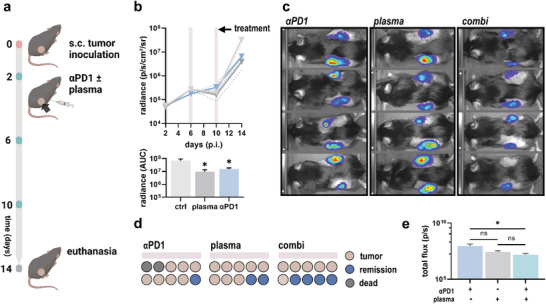
Medical gas plasma technology synergizes with anti‐PD1 immune checkpoint blockade in a syngeneic model of melanoma in vivo. a) Study protocol. b) Tumor bioluminescence 2, 6, 10, and 14 days after subcutaneous (s.c.) tumor inoculation and calculated area under the curve (AUC). Graphs show mean ± standard error of the mean (SEM). Statistical analysis was performed using one‐way analysis of variances (ANOVA) (*p < 0.05). c) Representative bioluminescent images. d) Remission rates based on bioluminescence. Each circle refers to an individual animal. e) Tumor bioluminescence on day 11. Bar graphs show mean ± SEM. Statistical analysis was performed using one‐way ANOVA (*p < 0.05). ns = nonsignificant. αPD1 = anti‐PD1. p.i. = postinoculation.

**Figure 5:**
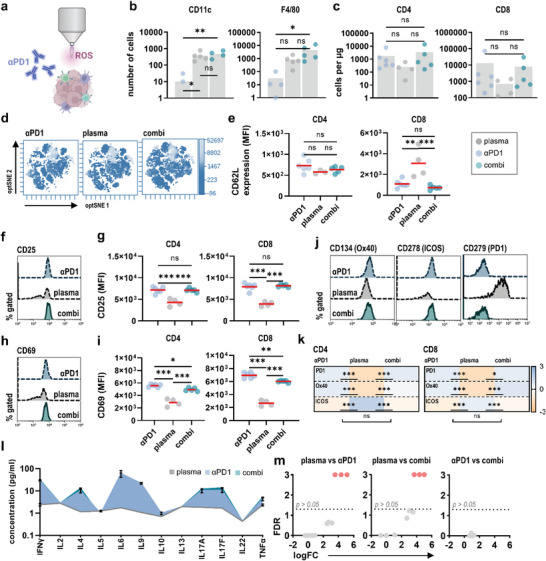
Medical gas plasma and anti‐PD1 immune checkpoint blockade synergize in remodeling the intratumoral melanoma immune landscape. a) Study protocol. b,c) Flow cytometric quantification of myeloid (b) and lymphoid (c) leucocytes in tumors retrieved from melanoma‐bearing animals. Bar graphs show mean and individual data points. Statistical analysis was performed using one‐way analysis of variances (ANOVA) (*p < 0.05, **p < 0.01). d,e) Representative optSNE plots of CD62L expression (d) and quantification thereof on CD4^+^ and CD8^+^ lymphocytes (e). Graphs show mean (line) and individual data points. Statistical analysis was performed using one‐way ANOVA (**p < 0.01, ***p < 0.001). f,g) Representative flow cytometry intensity histograms of CD25 expression (f) and quantification thereof on CD4^+^ and CD8^+^ lymphocytes (g). Graphs show mean (line) and individual data points. Statistical analysis was performed using one‐way ANOVA (***p < 0.001; g). h,i) Representative flow cytometry intensity histograms of CD69 expression (h) and quantification thereof on CD4^+^ and CD8^+^ lymphocytes (i). Graphs show mean (line) and individual data points. Statistical analysis was performed using one‐way ANOVA (*p < 0.05, **p < 0.01, ***p < 0.001; i). j,k) Representative flow cytometry intensity histograms of CD134 (Ox40), CD278 (ICOS), and CD279 (PD1) expression (j) and quantification thereof on CD4^+^ and CD8^+^ lymphocytes (k). Heat map shows mean. Statistical analysis was performed using one‐way ANOVA (*p < 0.05, ***p < 0.001; k). l) Intratumoral cytokines. Graph shows mean ± standard error of the mean (SEM). m) Volcano plots showing differentially expressed cytokines (*p* < 0.05). Statistical analysis was performed using one‐way ANOVA. ns = nonsignificant. αPD1 = anti‐PD1. ROS = reactive oxygen species. MFI = mean fluorescence intensity. FDR = false discovery rate. FC = fold change.

Before investigating the therapeutic effects of combined gas plasma–ICB, the efficiency of respective monotreatments was first confirmed in vivo. For tumor induction, 5 × 10^4^ syngeneic B16F10 melanoma cells were injected in both flanks of C57/Bl6 mice. Animals were left untreated, received intraperitoneal injection of anti‐PD1 checkpoint antibodies or local application of medical gas plasma in three treatment cycles starting from day 2 postinoculation (**Figure** **4**a). Tumor growth was monitored based on bioluminescent imaging showing that both medical gas plasma and anti‐PD1 ICB successfully reduced melanoma growth progression (Figure 4b). Next, monotherapies were tested against the combination of both (Figure 4c). Strikingly, combined gas plasma–ICB increased remission rates compared to anti‐PD1 and gas plasma monotreatments (Figure 4d) and reduced melanoma growth progression in tumor‐bearing animals (Figure 4e). Moreover, a tending contralateral reduction in growth of the non‐plasma‐treated tumor indicated an augmented abscopal effect (Figure S3). We hypothesized that the augmented efficacy of combined gas plasma–ICB would also correlate with an altered immune landscape in the TME. To this end, single‐cell suspensions were retrieved from each tumor and levels of tumor‐infiltrating leucocytes were evaluated (**Figure** **5**a). Flow cytometric analysis of individual tumors revealed gas plasma treatment to increase infiltration of CD11c^+^ dendritic cells and F4/80^+^ macrophages in the tumor bed, which was also observed upon combined ICB, but not in animals receiving ICB monotreatment (Figure 5b). By contrast, significant differences in CD4^+^ and CD8^+^ T cell infiltration could not be observed (Figure 5c). Regulation of CD62L (l‐selectin) expression plays a pivotal role in T cell trafficking to and from peripheral lymph nodes. Shedding of CD62L (Figure 5d), as a surrogate marker for T cell activation, was significantly increased on CD8^+^ cytotoxic T cells upon combined ICB and monotreatment (Figure 5e). Moreover, ICB was a major predictor for CD25 expression (Figure 5f) on CD4^+^ and CD8^+^ T cells (Figure 5g). Similar observations were made for CD69 (Figure 5h) underlining increased activation of CD4^+^ and CD8^+^ T cells upon exposure to ICB (Figure 5i). T cell exhaustion as a state of T cell dysfunction is characterized by increased expression of alternate checkpoints on T lymphocytes upon chronic activation and can impair response to ICB. To this end, surface expression of CD134 (Ox40), CD278 (ICOS), and CD279 (PD1) was evaluated on tumor‐infiltrating T cells using flow cytometry (Figure 5j). Interestingly, ICB reduced relative expression of Ox40 and (unbound) PD1 on intratumoral T cells, while ICOS expression was only reduced upon plasma monotreatment (Figure 5k). By mapping intratumoral cytokine release profiles using a bead‐based multiplex ELISA, we aimed to understand further the immunological consequences of combined gas plasma–ICB (Figure 5l). Antibodies targeting interferon gamma (IFNγ), tumor necrosis factor alpha (TNFα), and interleukin (IL) 2 indicated only minor differences in antitumoral T_H_1 responses when comparing the different treatment regimes. A slight increase was found for T_H_2‐ and T_H_17‐related cytokines, including IL4, IL6, IL17A, and IL17F in animals receiving ICB (Figure 5m).

### 2.4 Combinational Gas Plasma and Anti‐PD1 Checkpoint Therapy Alter Intratumoral Immune‐Related Gene Expression Signatures

**Figure 6:**
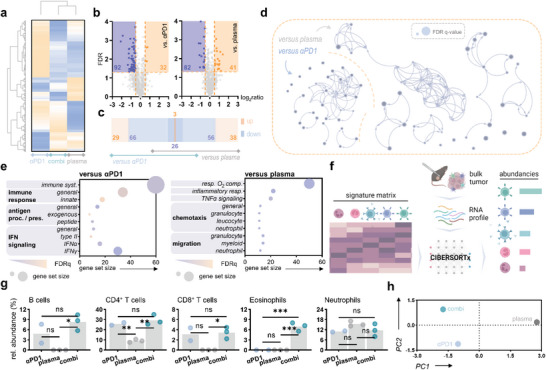
Combinational gas plasma and checkpoint therapy alter intratumoral immune‐related gene expression signatures. a) WPGMA‐weighted hierarchical clustering of gene expression levels in bulk RNA samples retrieved from melanoma‐bearing mice. b) Volcano plot showing differentially (*p* < 0.05) up‐ (orange) and downregulated genes (blue). c) Venn diagram showing overlapping differentially expressed genes (DEG). d) Cytoscape enrichment network of DEG. e) Pathway enrichment dot plots of gene ontology (GO) terms. f) log_2_ expression values of genes contributing to the GO term innate immune response. Graph shows mean ± standard error of the mean (SEM). g) Cell type abundancies were calculated from bulk RNA samples using the CIBERSORTx computational framework. Graph shows mean and individual data points. Statistical analysis was performed using one‐way analysis of variances (ANOVA) (*p < 0.05, **p < 0.01, ***p < 0.001). h) Principal component analysis (PCA) calculated from cell type abundances. ns = nonsignificant. αPD1 = anti‐PD1. FDR = false discovery rate. immune syst. = immune system. antigen proc./pres. = antigen procession and presentation. resp. O_2_ comp. = response to oxygen‐containing compound. rel. = relative. PC = principal component.

Immune‐related gene expression signatures were analyzed in bulk RNA samples derived from excised tumors of melanoma‐bearing mice using the NanoString murine PanCancer IO 360° Panel. WPGMA‐weighted hierarchical clustering outlined dissimilar expression patterns among the different treatment groups (**Figure** **6**a). Differentially expressed genes were subjected to further analysis (Figure 6b). In the Venn diagram, a concordant downregulation compared to ICB or plasma monotreatments is indicated for 26 targets. By contrast, an overlapping upregulation is shown for 3 targets, HES1, SOX10, and MLANA (Figure 6c). Gene set enrichment analysis (GSEA) revealed a strong interconnection of regulated processes (Figure 6d). Compared to ICB monotreatment, top‐regulated categories included processes related to innate immune responses, antigen processing and presentation, and IFN signaling upon plasma–ICB combination. By contrast, alterations in categories related to response to oxygen compounds, inflammatory responses, TNFα signaling, chemotaxis, and migration of myeloid cells were found compared to gas plasma monotreatment (Figure 6e). Notably, process category priority was the same for the different comparisons. Last, cell type abundancies were calculated from bulk RNA expression data using the CIBERSORTx computational framework (Figure 6f). Comparison of relative abundancies revealed a significant increase in B cell signatures compared to plasma monotreatment in the tumors. Along similar lines, eosinophil signatures were increased compared to both monotherapeutic approaches. In line with previous flow cytometric results, CD4^+^ and CD8^+^ T cell signatures were increased upon combined ICB and monotreatment (Figure 6g). Differences in intratumoral immune landscapes were underlined by a calculated PCA (Figure 6h).

## 3 Discussion

Strategies to improve therapeutic activity and selectivity are a major goal in oncological drug development. Driven by technological and diagnostical innovations, recent advances have been made by exploiting cancer‐associated molecular and environmental signatures using targeted drugs. Yet, acquired drug resistance and genome instability of cancer cells remain a major challenge of targeted therapies. Considering the genetic heterogeneity of cancers, it becomes evident that a combination of several agents might be required to effectively eliminate these cells. Here, the implementation of ROS‐based therapy approaches has been suggested.^[^ ^9^ ^]^ We investigated the ability of a novel anticancer treatment modality, medical gas plasma, to potentiate responses in therapeutic strategies targeting melanoma.

Starting from a library of 46 mitochondria‐targeted drugs, 11 drugs were identified showing strong synergistic effects upon combination with gas plasma‐mediated melanoma oxidation in two cell lines. Hereof, six drug combination partners were excluded due to superior toxicity in nonmalignant human keratinocytes, indicating an unfavorable risk‐benefit profile and a low therapeutic index. The remaining five combination regimes (A‐1210477, Carvedilol, Cozymasei, SBI‐0206965, and Navitoclax) were subjected to further analysis. Increased consumption of the antioxidant tripeptide glutathione indicated oxidative distress in melanoma cells, which was expected upon exposure to medical gas plasma as a redox‐based treatment modality but was not increased in drug monotreatment and combination regimes. Cell death modalities predominantly induced in combination regimes were evaluated using the pan‐caspase, hence apoptosis, inhibitor Z‐VAD‐FMK, and the RIP‐1 kinase inhibitor necrostatin‐1 (nec‐1) for inhibition of necroptosis. In the presence of Z‐VAD‐FMK but not nec‐1, a significant cell death inhibition was found in B16F10 and SK‐MEL‐28 melanoma cell lines, indicating apoptosis as the preferred mode of execution. Attributing ROS‐induced cell death to a certain modality is complicated not only by the heterogeneous and cell‐type‐specific death responses but further by the fact that exposure to exogenous (plasma‐derived) ROS can lead to endogenous ROS amplification, making it difficult to distinguish between responses to either. The notion that medical gas plasma triggers proapoptotic signaling in many cell types is emphasized by previous studies that observed an increase of proapoptotic Bax over Bcl‐2,^[^ ^24^, ^25^ ^]^ phosphatidylserine flipping,^[^ ^26^ ^]^ and DNA fragmentation.^[^ ^27^ ^]^ Yet, other pathways, including ferroptosis,^[^ ^28^, ^29^ ^]^ lysosome‐dependent cell death,^[^ ^30^, ^31^ ^]^ or autophagy,^[^ ^32^ ^]^ have been convincingly reported in the literature. In accordance with the Nomenclature Committee on Cell Death, apoptosis is defined as a form of regulated cell death initiated by perturbations of the intracellular or extracellular microenvironment, which is demarcated by mitochondrial outer membrane permeabilization (MOMP) and precipitated by executioner caspases, mainly caspase 3.^[^ ^33^ ^]^ Both characteristics, loss of mitochondrial membrane potential as a sign of MOMP paralleled by activation of two executioner caspases 3 and 7, were exhibited upon combined drug–plasma treatment. The superior toxicity of combined drug–plasma treatment was confirmed for all regimens in a syngeneic model of melanoma in vivo. Here, reduced tumor growth was accompanied by increased activation of effector caspase 3 and a reduced proliferative index. Cancer preventive and therapeutic effects have been prescribed for A‐1210477, Carvedilol, SBI‐0206965, and Navitoclax before. Hereof, a number of phase 1 and 2 clinical studies^[^ ^34^, ^35^, ^36^ ^]^ have been enrolled in evaluating the use of Navitoclax, a targeted high‐affinity inhibitor of Bcl‐2, in the treatment of lymphoid malignancies based on previous studies indicating a broad activity against a panel of human tumor cell lines in vitro and durable tumor regression in vivo.^[^ ^37^, ^38^, ^39^ ^]^ In preclinical studies, targeting antiapoptotic Bcl‐2 family proteins has likewise succeeded in using A‐1210477 in treating BRAF‐mutant melanoma. Our study indicates that ROS‐induced proapoptotic signaling synergizes with inhibitors of antiapoptotic protein families, potentiating their effects. SBI‐0206965 is a highly selective ULK1 kinase inhibitor that blocks autophagy.^[^ ^40^ ^]^ Elimination of damaged proteins, protein complexes, and organelles by autophagy is a central cellular mechanism and plays a crucial physiological role in response to endo‐ or exogenous stressors. However, in cancer, where cells experience ongoing metabolic stress, autophagy can promote tumor initiation, progression, and resistance to treatments, motivating its pharmacological inhibition.^[^ ^41^ ^]^ Hence, despite direct initiation of proapoptotic signaling partners, inhibition of cellular stress responses could potentiate ROS‐induced oxidative distress alike. Among the five selected compounds, carvedilol is the only clinically applied drug. Although FDA‐approved for treating cardiovascular diseases, recent evidence suggests that carvedilol use is associated with reduced cancer risk^[^ ^42^ ^]^ and has been implicated as a coadjuvant therapy.^[^ ^43^ ^]^ However, the mechanisms are not yet fully understood. Notwithstanding, additive or synergistic effects of combined drug–plasma treatment have been described before. For instance, plasma treatment has been shown to restore the chemosensitivity of TRAIL‐resistant colorectal cancer and chemoresistant multiple myeloma and glioblastoma.^[^ ^44^, ^45^, ^46^, ^47^ ^]^ In melanoma, responses to doxorubicin,^[^ ^48^, ^49^ ^]^ epirubicin, oxaliplatin, vorinostat,^[^ ^50^ ^]^ and dacarbazine have been observed.^[^ ^51^ ^]^ Moreover, the combination with radiotherapy^[^ ^52^ ^]^ and nanoparticles have improved responses in vitro and in vivo.^[^ ^53^, ^54^, ^55^ ^]^ However, this is the first study systematically investigating the synergistic effects of combined gas‐plasma treatment to identify eligible therapeutic targets for clinical application in dermato‐oncology.

Notably, novel therapies have to be efficient and safe. To this end, our screening approach encompassed exclusion of treatment regimens showing high toxicity in HaCaT keratinocytes, indicating a low therapeutic index. The safety of gas plasma treatment has been convincingly demonstrated in previous studies. Following kINPen plasma exposure, anti‐ but not prometastatic effects were observed in vitro.^[^ ^46^ ^]^ Genotoxic events were found to be absent using the WHO‐accredited hen's egg test for micronucleus induction model.^[^ ^56^ ^]^ A 1‐year follow‐up in mice receiving repeated plasma treatment of skin wounds showed a lack of gas plasma‐induced preneoplastic or neoplastic lesions after a 1‐year follow‐up.^[^ ^57^ ^]^ Similar observations have been made in a 5‐year follow‐up in a wound patient cohort lacking malignant transformation, inflammatory reactions, or pathological modifications after plasma treatment.^[^ ^58^ ^]^ Following repeated plasma exposure of the oral mucosae over 12 months, histopathological analysis of 406 animals did not indicate increased formation of noninvasive lesions or squamous cell carcinomas.^[^ ^59^ ^]^

Despite direct tumor toxic effects, our findings indicated immune‐modulatory aspects of combined drug–plasma treatment as seen by increased levels of tumor‐infiltrating leucocytes. Immune‐targeted therapies have been a major breakthrough in many malignancies in recent years.^[^ ^60^, ^61^ ^]^ While the majority of conventional chemotherapeutics elicit cytotoxicity by interfering with proteins affecting DNA synthesis and replication, immunotherapies aim to disrupt local and systemic immune perturbations occurring during malignant transformation to spark a self‐sustaining cycle of cancer immunity. However, despite valuable excitement dedicated to advances in antibody and cell‐based therapy approaches, immunotherapy still remains ineffective for the majority of patients with cancer.^[^ ^3^, ^62^ ^]^ Efficient priming of an antitumor immune response is often impaired by primary and secondary resistance mechanisms causing insufficient initiation of the cancer immunity cycle involving (a) antigen presentation and T cell activation, (b) infiltration of T cells in the tumor microenvironment, and (c) efficient T cell killing activity.^[^ ^63^ ^]^ Checkpoint inhibitors prove their advantages in “hot” tumors, given that the immune system has successfully passed the finely tuned process of antigen‐cross presentation and critical T cell priming in lymphoid tissues but will fail their effect without previous generation of antigen‐specific effector T cells. The limitations of ICB have pushed research in immuno‐oncology toward combinational treatments lately. Strikingly, shortly after the succession of nivolumab (anti‐PD1)–ipilimumab (anti‐CTLA4) as the first ICB combination treatment in advanced melanoma,^[^ ^64^ ^]^ pairing of pembrolizumab (anti‐PD1) and platinum‐based chemotherapy in nonsmall lung cancer was granted FDA‐approval in 2017.^[^ ^65^ ^]^ By eliciting an inflammatory response to dying cancer cells, a so‐called ICD, coadministered chemotherapeutics turned tumors from “cold” to “hot”, synergizing with ICB in unleashing a potent antitumor immune response. Furthermore, several studies demonstrated increased efficacy of combined radiotherapy and ICB, synergizing via activation of inflammatory pathways, induction of ICD, release of antigens from irradiated cells, and increased tumor sensitization to T cell responses.^[^ ^66^ ^]^ Experimental strategies for the detection of ICD encompass providing evidence of (a) expression and release of DAMPs on dying cancer cells followed by (b) successful activation of antigen‐presenting cells in vitro and (c) dying cancer cells to initiate adaptive immunity in a syngeneic, immunocompetent host in vivo.^[^ ^67^ ^]^ In preclinical plasma research, a range of surrogate ICD‐related DAMPs, including ecto‐expression of calreticulin and heat shock proteins,^[^ ^68^, ^69^ ^]^ release of ATP^[^ ^70^ ^]^ and nuclear translocation of HMGB‐1^[^ ^71^ ^]^ has been convincingly shown upon oxidation in many different cell lines. Likewise, chemokine detection in culture supernatants of plasma‐oxidized tumor cells revealed an increased release of several proimmunogenic and chemotactic agents.^[^ ^72^ ^]^ Functional analysis of APCs after challenging with plasma‐oxidized tumor cells increased phagocyte activation,^[^ ^73^ ^]^ followed by enhanced tumor material uptake^[^ ^74^ ^]^ as seen in our study and interaction with T cells.^[^ ^69^ ^]^ Moreover, a recent screening on a protein library pointed out to an inherent DAMP activity of oxidatively modified proteins generated by plasma treatment^[^ ^75^, ^76^ ^]^ comparable to advanced glycosylation end‐products on naturally occurring carbohydrates.^[^ ^77^ ^]^ Following successful cross‐presentation and T cell priming, an inhospitable TME may preclude proper function of the expanded T cell repertoire. This might include the aberrant expression of immune checkpoints or coinhibitory receptors, immunosuppressive cytokines, and immune‐inhibitory metabolites.^[^ ^78^ ^]^ Strikingly, evaluation of checkpoint expression signatures in vitro and in vivo in our study revealed an overall downregulation of investigated markers after plasma treatment.

In vivo, the ability of dying cancer cells to drive adaptive immunity depends both on their adjuvanticity and antigenicity. While the former is an inherent trait of most ICD inducers, stimulating the coordinated release and expression of danger signals necessary for the recruitment of APCs, this does not account for tumor cell antigenicity. Malignant transformation and cancer progression are frequently accompanied by a high mutation rate in cancer cells, culminating in MHC I‐mediated expression of tumor neoantigens. Representing a poor structural homology of physiologically occurring epitopes, neoantigens fail to induce clonal deletion in the context of central tolerance and can be recognized by T cells.^[^ ^79^, ^80^ ^]^ However, genetic and epigenetic defects, including antigen loss and subclonal evolution combined with impaired antigen presentation, often comprise adaptive immune responses.^[^ ^81^ ^]^ Therapeutic regimens that boost tumor antigenicity are hence considered to potentiate the ability of dying cancer cells to initiate an adaptive immune response.^[^ ^82^, ^83^ ^]^ Using gas plasma technology, post‐translational, oxidative modification of the model protein ovalbumin (oxOva) enhanced T cell activation compared to native ovalbumin (Ova) in genetically engineered mice recently. Moreover, oxOva vaccination of wild‐type mice provided enhanced protection from B16F10‐Ova melanoma growth, indicating a pronounced antitumor immune response in terms of an amplified and/or broadened T cell response.^[^ ^22^ ^]^ From those findings, it is hypothesized that plasma‐derived ROS create an inflammatory‐like environment able to trigger antitumor immune responses not only by increasing tumor cell adjuvanticity but also their antigenicity by formation of neoantigens via oxidation of biomolecules. Yet, the exact determinants are not fully understood. Concomitant with enhanced immune cell infiltration and leucocyte activation in melanoma‐bearing mice, we recently provided evidence of local plasma treatment to induce ICD‐mediated adaptive immunity and protect mice from tumor growth after preventive vaccination.^[^ ^84^ ^]^ Evidence of medical gas plasma technology to drive adaptive immunity in vivo as the ultimate proof of ICD has further been supported by reports on abscopal effects after plasma‐mediated tumor oxidation in murine tumor models recently.^[^ ^85^, ^86^, ^87^ ^]^ In this light, we hypothesized that combined gas plasma treatment could potentiate responses to anti‐PD1 ICB, which was confirmed in a syngeneic model of melanoma in vivo. Interestingly, flow cytometric analysis of the intratumoral immune compartment revealed a major influence of plasma treatment on the innate immune compartment, while effects on the lymphoid immune compartment were dominated upon checkpoint administration. This was emphasized by evaluation of gene expression profiles in bulk RNA tumor samples based on the NanoString PanCancer IO 360 Panel. Compared to ICB monotreatment, GSEA indicated alterations in innate immune responses, antigen presentation and processing, and IFN response. In line with previous flow cytometric results, cell type abundancies characterized from bulk RNA samples using the CIBERSORT computational framework indicated an increase in CD4^+^ and CD8^+^ T cell signatures in animals receiving checkpoint administration. An additional increase in expression signatures associated with B cell infiltration was observed upon combined plasma–ICB compared to plasma monotreatment. Along similar lines, eosinophil signatures were increased compared to both monotherapeutic approaches. Our data indicate that local tumor oxidation using gas plasma technology primarily acted on APCs which may facilitate the generation of antigen‐specific T cell responses. Combining medical gas plasma treatment with anti‐PD1 checkpoint therapy suppressed tumor growth more effectively than either agent alone. We hypothesize that by promoting the successful initiation of the cancer immunity cycle, as discussed above, plasma treatment provides the fundamental basis for efficient ICB, unleashing efficient T cell killing activity only after the previous generation of a pool of antigen‐specific effector T cells. Moreover, we found plasma treatment to reduce the aberrant expression of coinhibitory surface markers, supporting the proper function of the expanded T cell repertoire. Clinically, the dual application of gas plasma and ICB in cancer therapies could broaden the range of patients profiting from ICB. As a major advantage, plasma treatment is an easy‐to‐implement, cost‐effective technology with good tolerability, already applied in many dermatological centers in Europe. However, it should be mentioned that the treatment is limited to superficially growing skin cancers for now, as eligible plasma sources for treating internal neoplasms have not yet been developed.

In summary, this study systematically demonstrates the ability of gas plasma‐mediated tumor oxidation to potentiate drug responses in the treatment of melanoma. From our findings, it can be concluded that agents targeting stress responses (e.g., autophagy inhibitors), antiapoptotic proteins (e.g., bcl‐2), or immune‐targeted therapies may particularly profit from such an approach.

## 4 Conclusion

Implementation of ROS‐based therapy approaches in therapeutical combination regimes has been suggested to potentiate therapeutic responses in oncology. We hypothesized that combined gas plasma‐mediated tumor oxidation could potentiate drug responses in the treatment of melanoma and aimed to identify eligible agents in a systematic screening approach. Starting from a library of 46 mitochondria‐targeted drugs, five drugs were identified to synergize with plasma treatment in vitro and in vivo. The systematic screening indicated that plasma treatment might potentiate responses to inhibitors of antiapoptotic protein families and inhibitors of cellular stress responses, outlining eligible target mechanisms to identify further combination partners in future studies. In addition, immunomodulatory aspects of the treatment were found in vivo, motivating us to investigate if combined gas plasma treatment could further potentiate responses to anti‐PD1 ICB. Strikingly, combining gas plasma treatment with anti‐PD1 ICB suppressed tumor growth more effectively than either agent alone. Evaluation of underlying mechanisms indicated that local tumor oxidation using gas plasma technology primarily acted on APCs. We hypothesize that plasma treatment provides the fundamental basis for efficient ICB by promoting the successful initiation of the cancer immunity cycle. Collectively, our results indicate that gas plasma treatment could be a powerful tool to sensitize cancer cells to a broad range of oncological treatment approaches in the future.

## 5 Experimental Section

### Cell Culture

Human SK‐MEL‐28 (CLS: 300337; CLS cell line service, Germany), MaMel‐86a (RRID: CVCL_A221), A375 (ATCC: CRL‐1619; ATCC, Germany), and murine B16F10 (ATCC: CRL‐6475; ATCC, Germany) melanoma cells were cultured in Dulbecco's modified Eagle or Roswell Park Memorial Institute (both from PanBiotech, Germany) medium supplemented with 10% fetal bovine serum, 1% l‐glutamine, and 1% streptomycin (all Corning, Germany) according to the supplier's instructions. Human HaCaT (CLS: 300493; CLS cell line service, Germany) keratinocytes were cultured in Roswell Park Memorial Institute medium supplemented with 10% fetal bovine serum, 1% l‐glutamine, and 1% streptomycin. Cells were kept under standard culture conditions at 37 °C, 5% CO_2_, and 95% humidity in a specified cell culture incubator (CB212; Binder, Germany).

### Medical Gas Plasma Jet Technology

Gas plasma treatment in this study was performed using an atmospheric pressure plasma jet (kINPen; neoplas, Germany). The device is technically similar to the kINPen Med, which has been accredited as medical class IIa/IIb in Europe since 2013 and frequently applied in dermatology.^[^ ^88^ ^]^ In standard mode, the jet is operated using a flow of argon gas (purity 99.9999%; Air Liquide, France) at three standard liters per minute (slm). The noble gas was excited at the electrode within the head of the plasma jet at a frequency of 1 MHz at a generated power of ≈1 W. The jet was installed on an *xyz* motorized table (CNC step) controlled via software to maximize the reproducibility of the plasma treatment.

### Intracellular ROS/RNS

Cells were stained with 2 µm 2′,7′‐dichlorodihydrofluorescein diacetate (DCF‐DA; Thermo Fisher, Germany) for 30 min at 37 °C prior to plasma treatment. After washing, cells were exposed to plasma and 1 µm 4′,6‐Diamidin‐2‐phenylindol (DAPI; Sigma‐Aldrich, Germany) was added immediately to stain nuclei of terminally dead cells. Images were acquired in brightfield and fluorescence channels at *λ*_ex_ 365 nm and *λ*_em_ 465 nm for DAPI and *λ*_ex_ 475 nm and *λ*_em_ 525 nm for DCF using High Content Imaging (Operetta CLS; PerkinElmer, Germany) and a 20× air objective (NA = 0.4; Zeiss, Germany).

### In Vitro Screening Study

A library of 46 mitochondria‐targeted compounds (Cat# L5300; TargetMol, USA; Table S1, Supporting Information) was screened for combination effects with medical gas plasma technology. For in vitro experiments, 5 × 10^3^ cells were seeded in 96‐well flat bottom plates (Thermo Fisher, Germany) 24 h prior to experiments. The cell culture medium was refreshed on the day of experiments. A three‐step screening strategy (Figure 1a) was employed to identify promising drug candidates. First, cells were exposed to 0.01, 0.1, 1, and 10 µm of each compound to generate dose–response curves based on cellular metabolic activity. Cells exposed to plasma only received dimethyl sulfoxide (DMSO) instead of drug treatment. Gas plasma treatment was done at 2 slm for 30 s 16 h after exposure to the respective compounds or DMSO in plasma monotreatment and combination groups, respectively. Drugs showing significant combination effects were selected. The coefficient of drug interaction (CDI) was calculated to evaluate if the observed effect was additive or synergistic based on the formula: CDI = AB/(A × B). Second, IC50 values were calculated for drug monotreatment and combination groups. Drugs showing a fivefold (FC > 5) decreased IC50 value in combination regimes were tested on nonmalignant HaCaT keratinocytes in a similar fashion to determine the selectivity of the approach. Functional analysis was performed on drugs showing a twofold (FC > 2) decreased IC50 in SK‐MEL‐28 human melanoma cells compared to nonmalignant HaCaT keratinocytes.

### Metabolic Activity

The Almar Blue assay was used to assess the metabolic activity of cells. Briefly, 100 µm 7‐hydroxy‐3H‐phenoxazin‐3‐on‐10‐oxid (resazurin; Alfa Aesar, USA) was added to the cells 24 h after treatment, followed by 3 h of incubation under standard culture conditions. Viable cells metabolize nonfluorescent resazurin to fluorescent resorufin in an NADPH/H^+^‐dependent reaction. Resorufin fluorescence was measured at *λ*_ex_ 535nm and *λ*_em_ 590 nm using a multimode plate reader (F200; Tecan, Switzerland).

### Cellular Viability

Cellular viability was assessed using high‐content imaging 6 and 24 h after exposure. Briefly, cells were stained with sytox green (SG) for 30 min at 37 °C and cell culture media was exchanged immediately after to reduce autofluorescence. Images were acquired in brightfield and fluorescence channels at *λ*_ex_ 490 nm and *λ*_em_ 520 nm using a 20× air objective (NA = 0.4; Zeiss, Germany). Algorithm‐driven image analysis was performed using Harmony 4.9 image analysis software (PerkinElmer; Germany). The analysis sequence included cellular segmentation based on digital phase contrast (DPC), and cellular viability was assessed based on SG fluorescence.

### Intracellular Glutathione

Intracellular levels of reduced GSH as a surrogate marker for cellular redox capacities were determined using High Content Imaging (Operetta CLS; PerkinElmer, Germany). Briefly, cells were stained with 5 µm GSH‐tracer (Tocris, UK) for 90 min at 37 °C 6 h after treatment. The excitation/emission maxima of the cell‐permeable, ratiometric probe shift from *λ*_ex_/*λ*_em_ 520/580 nm to *λ*_ex_/*λ*_em_ 430/510 nm upon binding to reduced GSH. The F_510_/F_580_ ratio correlates with the intracellular GSH concentration. After washing, images were acquired in brightfield, DPC (pseudocytosolic signal) and fluorescence channels for bound (*λ*_ex_ 390–420 nm and *λ*_em_ 500–550 nm) and unbound (*λ*_ex_ 460–790 nm and *λ*_em_ 570–650 nm) using a 20× air objective (NA = 0.4; Zeiss Germany). The experimental setup and the software‐based quantification algorithms were done using Harmony 4.9 image analysis software (PerkinElmer, Germany). Cells were detected via DPC signal for image segmentation, and the mean fluorescence intensity (MFI) of bound and unbound GSH tracer was quantified. Intracellular levels of reduced GSH were calculated according to the formula: GSH level = (MFI [bound tracer])/(MFI [unbound tracer]).

### Mitochondrial Dysfunction

Mitochondrial dysfunction was evaluated 24 h after treatment using High Content Imaging (Operetta CLS; PerkinElmer, Germany). Briefly, cells were loaded with 100 nm tetramethylrhodamine ethyl ester (TMRE) for 30 min at 37 °C. TMRE is a cell‐permeable, cationic fluorescent dye readily sequestered by active mitochondria. After washing, images were acquired in brightfield, DPC and fluorescence channels at *λ*_ex_ 550 nm and *λ*_em_ 580 nm using a 20× air objective (NA = 0.4; Zeiss, Germany). Algorithm‐driven image analysis was performed using Harmony 4.9 image analysis software (PerkinElmer, Germany). Cells were detected via DPC signal for image segmentation, and TMRE mean fluorescence intensity was quantified.

### Cell Death Inhibition and Activation of Effector Caspases

Cell death modalities were investigated using respective inhibitors. For inhibition of apoptosis, cells were loaded with 50 µm of the cell‐permeable, pan‐caspase inhibitor Z‐VAD‐FMK 1 h prior to plasma treatment. Likewise, Necrostatin‐1 (nec‐1) was used for necroptosis inhibition at a final concentration of 40 µm. Cellular viability was evaluated using High Content Imaging (Operetta CLS; PerkinElmer, Germany) as described above. Therefore, cells were stained with sytox orange for 30 min at 37 °C and cell culture media was exchanged immediately after to reduce autofluorescence. Initiation of apoptosis was further evaluated based on activation of the effector caspases 3 and 7. Briefly, cells were stained with Cell Event caspase 3/7 reagent (Thermo Fisher, Germany) at a concentration of 400 nm for 15 min at 37 °C 24 h after treatment. After washing, cells were acquired using flow cytometry (CytoFLEX S; Beckman‐Coulter, Germany). Analysis was done using Kaluza 2.2 software (Beckman‐Coulter, Germany).

### 5.1 3D Melanoma Spheroids

Human SK‐MEL‐28 cells were seeded at a density of 1 × 10^4^ cells per well in 96‐well ultralow attachment plates (PerkinElmer, Germany) and centrifuged at 1000 × *g* for 10 min. Consistent 3D melanoma spheroids formed after 48 h followed by drug, plasma, or combined treatment as described before. Spheroid cytotoxicity was evaluated using High Content Imaging (Operetta CLS; PerkinElmer, Germany) 24 h after exposure. Briefly, spheroids were stained with 5 µm SG for discrimination of terminally dead cells and 40 µm Hoechst (both Thermo Scientific, Germany), for nuclear staining, for 1 h at 37 °C. Immediately after, images were acquired in brightfield and fluorescence channels at *λ*_ex_ 490 nm and *λ*_em_ 520 nm for SG and *λ*_ex_ 365 nm and *λ*_em_ 465 nm for Hoechst from a z‐stack of 30 images at a 10 µm distance using a 5× air objective (NA = 0.16; Zeiss, Germany). Algorithm‐driven image analysis was performed using Harmony 4.9 image analysis software (PerkinElmer, Germany).

### In Vivo Antimelanoma Drug Combination Therapy

The combinational efficacy of promising drug candidates identified in vitro drug screenings was further investigated in a syngeneic model of melanoma in vivo. The ethical implications for the experiment were reviewed by the local ethical authority Comissão de Ética no Uso de Animais (CEUA/UEL) of the University of Londrina, Brazil (approval number: 3185.2019.53). Wildtype C57BL/6 mice were shaved on the left flank and subcutaneously inoculated with 1 × 10^6^ B16F10 syngeneic murine melanoma cells in PBS. Animal randomization was done once the tumor volume reached ≈50 mm^3^. Animals were divided into ten groups (7 animals per group), including one vehicle group (DMSO), one group exposed to gas plasma (3 slm of argon, 5 min), four groups receiving intraperitoneal injection of respective drugs (A‐1210477, Carvedilol, SBI‐0206965 or Navitoclax; all at 10 mg kg^−1^), and four groups receiving combined drug–plasma treatment. In combination groups, intraperitoneal drug injection was done 1 h prior to gas plasma treatment. Animals received treatments in four treatment cycles every second day. Animals were euthanized 24 h after the last treatment cycle. Tumors were carefully excised, and tumor weight was assessed using a precision balance. Excised tumors were embedded in optimal cutting temperature (Thermo Scientific, USA) compound and frozen in liquid nitrogen before storage at −80 °C.

### Immunohistochemistry

For immunohistochemistry, 5 µm sections were mounted on silanized slides, deparaffinized, rehydrated, immersed in 15 mmol L^−1^ citrate buffer (pH 6.0), and subjected to head‐induced epitope retrieval using a vapor lock for 40 min. The slides were rinsed with PBS, and peroxidase blockade was done using methanol/H_2_O_2_. Nonspecific protein binding was blocked with 3% bovine serum albumin. The sections were incubated overnight with primary antibodies targeting Ki‐67 (Santa Cruz, USA), cleaved caspase 3 (Cell Signaling, Germany), and CD45 (Santa Cruz, USA). After incubation, peroxidase‐conjugated secondary anti‐rabbit IgG antibodies (Santa Cruz, USA) were added for 1 h after washing. For chromogenic detection, slides were incubated with 3,3′‐diaminobenzidine for 5 min after washing and counterstained with hematoxylin. Negative controls were done by omitting the primary antibody. Images were acquired (Observer Z.1; Carl Zeiss, Germany), and quantification was done using ImageJ software.

### Flow Cytometry Immune Checkpoint Analysis

For in vitro immune checkpoint expression analysis, MaMel‐86a human melanoma cells were gas plasma exposed, cultured for 24 h, collected using accutase, and stained with several fluorescently labeled antibodies targeting immuno‐stimulating and suppressing receptors and ligands. After washing, cells were acquired using flow cytometry (CytoFLEX S; Beckman‐Coulter, Germany). Checkpoint expression signatures were further investigated on B16F10 tumors after in vivo exposure to plasma, anti‐PD1 ICB and combined plasma–ICB. Single‐cells suspensions retrieved from excised tumors were stained with antibodies targeting (conjugate) MHCI (BUV661), CD47 (PerCp‐Cy5.5), CD80/CD86 (APC), and CD278 (PE‐Cy7). DAPI (Sigma‐Aldrich, Germany) and iFluor maleimide 860 (AAT Bioquest, USA) were added for live–dead discrimination. After washing, cells were acquired using flow cytometry (CytoFLEX LX; Beckman‐Coulter, Germany), analysis was performed using Kaluza 2.2 software (Beckman‐Coulter, Germany).

### Peripheral Blood Mononuclear Cell (PBMC) Isolation and Generation of Monocyte‐Derived Dendritic Cells (DCs)

Isolation of PBMCs in this study was approved by the local ethics committee at the University Medical Center (approval number: BB 014‐14). PBMCs were isolated from buffy coats of healthy donors provided by the Institute of Transfusion Medicine Greifswald by density‐gradient centrifugation using lymphocyte separation medium (VWR, Germany). Residual erythrocytes were lysed using a red blood cell lysis buffer (BioLegend, Germany). For monocyte isolation, CD14 microbeads (Miltenyi Biotec, Germany) were used according to the manufacturer's protocol. Flow cytometric (Attune NxT; Applied Biosystems, USA) verification of purity was performed immediately after isolation and was always greater than 85%. For differentiation of monocytes into DCs, 1 × 10^5^ monocytes per well were seeded in 24‐well plates (Sarstedt, Germany). The cell culture medium was supplemented with 800 IU granulocyte‐macrophage colony‐stimulating factor (GM‐CSF) and 500 IU interleukin (IL) 4 (all PeproTech, Germany) to initiate monocyte‐derived dendritic cells (DCs). After two days, cells were restimulated by adding the same amount of GM‐CSF and IL‐4 to the cell culture medium. Immature DCs were used for coculture experiments on day 5.

### Phagocytosis of Oxidized Melanoma Cells

Human A375 melanoma cells were labeled with 1 µm CTV (Thermo Fisher, Germany) and seeded at a density of 1 × 10^5^ cells per well in 24‐well plates (Sarstedt, Germany). Immediately after, cells were exposed to plasma for 30 s. Oxidized melanoma cells were collected and added to immature DCs prepared from PBMCs, as described before. After incubation for 24 h, cocultured cells were harvested and stained with monoclonal antibodies (conjugate) targeting human CD11c (phycoerythrin‐cyanine 7; BioLegend, The Netherlands). For live–dead discrimination, iFluor maleimide 860 (AAT Bioquest, USA) was added. After washing, cells were acquired using flow cytometry (CytoFLEX LX; Beckman‐Coulter, Germany), analysis was performed using Kaluza 2.2 analysis software (Beckman‐Coulter, Germany). Tumor cells were identified by CTV staining, while DCs were distinguished based on CD11c expression.

### In Vivo Antimelanoma ICB and Plasma Treatment

The ethical implications for the experiment were reviewed by the local ethical authority Landesamt für Landwirtschaft, Lebensmittelsicherheit und Fischerei (LALLF) Mecklenburg Vorpommern (approval number: M‐V 7221.3‐1‐023/17). Wildtype C57BL/6 mice were shaved on the flank and subcutaneously inoculated with 5 × 10^4^ B16F10‐luc (PerkinElmer: BW124734; PerkinElmer, Germany) syngeneic murine melanoma cells in 100 µL of matrigel (Corning, Germany). First, treatment efficacies of monotreatments were confirmed. Animals received three treatment cycles of either (a) intraperitoneal injection of anti‐PD1 antibodies (clone: 29F.1A12; BioLegend, Germany), (b) 5 min local gas plasma treatment (kINPen Med; neoplas, Germany; 3 slm argon) or (c) were left untreated (control). Tumor growth was monitored using bioluminescence imaging (IVIS; PerkinElmer, Germany) over a time period of 20 min after intraperitoneal injection of 100 µL 30 mg mL^−1^ d‐luciferin (PerkinElmer, Germany). Animals were sacrificed on day 14 after tumor inoculation. Next, the combination regime was compared to monotreatment of anti‐PD1 and local gas plasma treatment. Animals received three treatment cycles of either (a) anti‐PD1 (clone: 29F.1A12), (b) 5 min local gas plasma treatment (kINPen Med; 3 slm argon) or (c) the combination of both. Again, tumor growth was monitored using bioluminescence imaging as described before. Single‐cell suspensions of excised tumors were retrieved using the GentleMacs tumor dissociation kit mouse and the OctaMacs device (both Miltenyi, Germany). Single‐cell suspensions were subjected to further analysis by flow cytometry to evaluate tumor‐infiltrating leucocytes. In addition, supernatants were collected after tumor dissociation and stored at −80 °C for intratumoral chemokine and cytokine analysis.

### Tumor‐Infiltrating Leucocytes

Abundancies and expression signatures of tumor‐infiltrating leucocytes were assessed using flow cytometry. Single‐cell suspensions retrieved from excised tumors were stained with two core panels. Tumor infiltrating CD4^+^ and CD8^+^ T cells were analyzed using antibodies targeting (conjugate) B220/45R, IA/IE, Ly6G, CD11c, F4/80 (DUMP; all APC‐Cy7), CD134 (APC), CD4 (AF700), CD62L (PE/Dazzle594), CD278 (PC7), CD279 (PE), CD45 (PerCP‐Cy5.5), CD3 (BV421), CD8 (BV510), CD69 (BV650), CD25 (BV785), and MHCI (BUV661). For live–dead discrimination, iFluor maleimide 860 (AAT Bioquest, USA) was added. After washing, cells were acquired using flow cytometry (CytoFLEX LX; Beckman‐Coulter, Germany), analysis was performed using Kaluza 2.2 software (Beckman‐Coulter, Germany). In a second core panel, the number of tumor‐infiltrating antigen‐presenting cells was assessed using monoclonal antibodies targeting (conjugate) CD206 (BV421), Ly6G (BV510), CD11b (BV605), IA/IE (PerCP‐Cy5.5), CD80/CD86 (PE), Ly6C (PE/Dazzle594), F4/80 (PE‐Cy7), Cd274 (APC), CD11c (AF700), and CD45 (APC‐Cy7). For live–dead discrimination, SG (Thermo Fisher, Germany) was added. After washing, cells were acquired using flow cytometry (CytoFLEX S and LX; Beckman‐Coulter, Germany).

### Chemokine and Cytokine Analysis

Intratumoral chemokines and cytokines were analyzed using a bead‐based sandwich multianalyte assay (LegendPLEX; BioLegend, The Netherlands) according to the manufacturer's instructions. The assay panel contained beads targeted against IFNγ, IL2, IL4, IL5, IL6, IL9, IL10, IL13, IL17A, IL17F, IL22, and TNFα. Beads were labeled with fluorescent detection antibodies, and samples were acquired using flow cytometry (CytoFLEX LX; Beckman‐Coulter, Germany). Absolute concentrations of respective analytes were calculated against a standard curve using the LEGENDplex data analysis software (Vigene Tech, USA).

### RNA Isolation and NanoString Gene Expression Analysis

RNA was isolated from excised tumors after generation of single‐cell suspensions using an RNA isolation kit (Bio & Sell, Germany) according to the supplier's protocol. The RNA concentration of each sample was determined using fluorescent RNA labeling (Quant‐it RiboGreen RNA Reagent; Thermo Fisher, Germany) and a fluorometer (DS‐11 FX; DeNovix, Germany). Absolute concentrations were calculated against a standard curve. Subsequent gene expression analysis was performed using the NanoString nCounter technology, which directly evaluates RNA levels without prior complementary DNA synthesis. Briefly, 50 ng RNA per sample was hybridized for 24 h at 65 °C with reporter and capture ProbeSets of the nCounter PanCancer IO 360 Panel targeting 770 genes specifically designed for research in immuno‐oncology. Immediately after, samples were loaded into the nCounter SPRINT cartridge and processed by the NanoString nCounter system. Differential gene expression analysis was performed using the nSolver 4.0 analysis software (all NanoString Technologies, USA). Pathway enrichment analysis was evaluated using GSEA Software^[^ ^89^, ^90^ ^]^ and processed with Cytoscape.^[^ ^91^ ^]^ Cell fractions were quantified from bulk tissue gene expression profiles using CIBERSORTx.^[^ ^92^ ^]^

### Statistical Analysis

Graphing and statistical analysis were done using prism 9.5.1 (GraphPad Software, USA). Statistical analysis was performed using *t*‐test or analysis of variances (ANOVA) where appropriate. Data show mean ± standard error of the mean if not indicated otherwise in the figure legends.

## Conflict of Interest

The authors declare no conflict of interest.

## Supporting information

Supporting Information

## Data Availability

The data that support the findings of this study are available from the corresponding author upon reasonable request.
