# Supplementary material for: Medical Gas Plasma Technology Combines with Antimelanoma Therapies and Promotes Immune‐Checkpoint Therapy Responses
Source: Adv Sci (Weinh). 2023 Aug 4;10(28):2303183. doi: 10.1002/advs.202303183 (PMC10558686; doi:10.1002/advs.202303183)
Supplement: Supplementary file 1 — Supporting Information [file ADVS-10-2303183-s001.pdf]

ADVANCED SCIENCE
Open Access

Supporting Information

for *Adv. Sci.*, DOI 10.1002/advs.202303183

Medical Gas Plasma Technology Combines with Antimelanoma Therapies and Promotes Immune-Checkpoint Therapy Responses

*Lea Miebach, Gabriella Melo-Zainzinger, Eric Freund, Ramona Clemen, Alessandra Lourenco Cecchini and Sander Bekeschus**

Supporting data

Medical gas plasma technology combines with anti-melanoma therapies and promotes immune-checkpoint therapy responses

§Lea Miebach[1,2], §Gabriella Melo-Zainzinger[2,3], #Eric Freund[1,2,4], #Ramona Clemen[2], Alessandra Lourenco Cecchini[5], *Sander Bekeschus[2,6]

1 Department of General, Thoracic, Vascular, and Visceral Surgery, Greifswald University Medical Center, Greifswald, Germany
2 ZIK *plasmatis*, Leibniz Institute for Plasma Science and Technology (INP), Greifswald, Germany
3 Cancer Research Unit, Boehringer Ingelheim, Vienna, Austria
4 Department of Neurosurgery, Wien University Medical Center, Vienna, Austria
5 Laboratory of Molecular Pathology, State University of Londrina, Londrina, Brazil
6 Clinic for Dermatology and Venereology, Rostock University Medical Center, Rostock, Germany

§ equally contributed as first authors
# equally contributed as second authors
* correspondence: sander.bekeschus@inp-greifswald.de

Keywords: B16F10; CAP; immuno-oncology; reactive oxygen species; ROS

Table S1. List of 46 mitochondria-targeted compounds used in this study. Drugs showing significant, synergistic effects but were excluded due to high toxicity in HaCaT keratinocytes are highlighted in orange. Promising drug candidates chosen for further functional analysis are highlighted in green.

Drug name	Short	CAS	Molecular formula	Mol.wt.	Target
3.6'-Disinapoyl sucrose	*Dis*	*139891-98-8*	$C_{34}H_{42}O_{19}$	*754.68*	*LDH release inhibitor*
**A-1210477**	**A12**	**1668553-26-1**	$C_{46}H_{55}N_7O_7S$	**850.04**	**MCL1 inhibitor**
ABT-199	*ABT1*	*1257044-40-8*	$C_{45}H_{50}ClN_7O_7S$	*868.44*	*Bcl-2 inhibitor*
ABT-737	*ABT7*	*852808-04-9*	$C_{42}H_{45}ClN_6O_5S_2$	*813.43*	*Bcl-2 inhibitor*
**Amlodipine**	**Aml**	**88150-42-9**	$C_{20}H_{25}ClN_2O_5$	**408.88**	**Calcium Channel inhibitor**
Blebbistatin	*Ble*	*856925-71-8*	$C_{18}H_{16}N_2O_2$	*292.33*	*ATPase inhibitor*
Brefeldin A	*Bre*	*20350-15-6*	$C_{16}H_{24}O_4$	*280.36*	*ATPase inhibitor*
BTB06584	*BTB*	*219793-45-0*	$C_{19}H_{12}ClNO_6S$	*417.82*	*ATPase inhibitor*
**Carvedilol**	**Car**	**72956-09-3**	$C_{24}H_{26}N_2O_4$	**406.47**	**Adrenergic Receptor antagonist**
Carvedilol phosphate	*CarP*	*610309-89-2*	$C_{24}H_{26}N_2O_4\cdot H_2O\cdot H_3O_4P$	*522.49*	*Adrenergic Receptor antagonist*
Ciclopirox	*Cic*	*29342-05-0*	$C_{12}H_{17}NO_2$	*207.27*	*ATPase inhibitor*
Ciclopirox ethanolamine	*CicEth*	*41621-49-2*	$C_{14}H_{24}N_2O_3$	*268.35*	*ATPase inhibitor*
Cinobufagin	*Cin*	*470-37-1*	$C_{26}H_{34}O_6$	*442.55*	*ATPase inhibitor*
**Cozymasei (NAD+)**	**Coz**	**53-84-9**	$C_{21}H_{27}N_7O_{14}P_2$	**663.43**	**Dehydrogenase**
Dexibuprofen	Dex	51146-56-6	$C_{13}H_{18}O_2$	206.28	Plasminogen inhibitor
Diazoxide	*Dia*	*364-98-7*	$C_8H_7ClN_2O_2S$	*230.67*	*ATPase inhibitor;*
Digoxin	*Dig*	*20830-75-5*	$C_{41}H_{64}O_{14}$	*780.96*	*ATPase inhibitor*
Docetaxel	*Doc*	*114977-28-5*	$C_{43}H_{53}NO_{14}$	*807.88*	*Microtubule-associated inhibitor*
Docetaxel trihydrate	*DocTri*	*148408-66-6*	$C_{43}H_{59}NO_{17}$	*861.95*	*Bcl-2 inhibitor*
Eniporide	*Eni*	*176644-21-6*	$C_{14}H_{16}N_4O_3S$	*320.37*	*ATPase inhibitor*
Esomeprazole Magnesium	*EsoMag*	*161973-10-0*	$C_{34}H_{36}MgN_6O_6S_2$	*713.12*	*ATPase inhibitor*
Esomeprazole Sodium	*EsoSod*	*161796-78-7*	$C_{17}H_{18}N_3O_3S\cdot Na$	*367.4*	*ATPase inhibitor*
**Etidronate**	**Eti**	**2809-21-4**	$C_2H_8O_7P_2$	**206.03**	**ATPase inhibitor**
Gambogic Acid	*GamAc*	*2752-65-0*	$C_{38}H_{44}O_8$	*628.75*	*Bcl-2 inhibitor*
Gossypol acetic acid	*GosAc*	*12542-36-8*	$C_{32}H_{34}O_{10}$	*578.61*	*Bcl-2 inhibitor*
HA14-1	*HA1*	*65673-63-4*	$C_{17}H_{17}BrN_2O_5$	*409.23*	*Bcl-2 inhibitor*
Ibuprofen	*Ibu*	*15687-27-1*	$C_{13}H_{18}O_2$	*206.28*	*COX inhibitor*
**Idebenone**	**Ide**	**58186-27-9**	$C_{19}H_{30}O_5$	**338.45**	**ROS inhibitor**
K858	*K85*	*72926-24-0*	$C_{13}H_{15}N_3O_2S$	*277.34*	*KSP inhibitor*
**Lonidamine**	**Lon**	**50264-69-2**	$C_{15}H_{10}Cl_2N_2O_2$	**321.17**	**Hexokinase inhibitor**
MRT67307	*MRT*	*1190378-57-4*	$C_{26}H_{36}N_6O_2$	*464.62*	*TBK1 inhibitor*
**Navitoclax**	**Nav**	**923564-51-6**	$C_{47}H_{55}ClF_3N_5O_6S_3$	**974.61**	**Bcl-2 inhibitor**
NU2058	*NU2*	*161058-83-9*	$C_{12}H_{17}N_5O$	*247.3*	*CDK inhibitor*

Obatoclax Mesylate	*ObaMes*	*803712-79-0*	$C_{20}H_{19}N_3O{\cdot}CH_4O_3S$	*413.49*	*Bcl-2 inhibitor*
Omecamtiv mecarbil	*OmeMec*	*873697-71-3*	$C_{20}H_{24}FN_5O_3$	*401.43*	*ATPase activator*
**Ouabain octahydrate**	**Oua**	**11018-89-6**	$C_{29}H_{44}O_{12}{\cdot}8H_2O$	**728.77**	**ATPase inhibitor**
Paclitaxel	*Pac*	*33069-62-4*	$C_{47}H_{51}NO_{14}$	*853.92*	*Microtubule-associated inhibitor; Bcl2 inhibitor*
Pantoprazole Sodium Hydrate	*Pan*	*164579-32-2*	$C_{16}H_{14}F_2N_3NaO_4S{\cdot}3/2H_2O$	*432.37*	*ATPase inhibitor*
PF 03716556	*PF0*	*928774-43-0*	$C_{22}H_{26}N_4O_3$	*394.48*	*ATPase inhibitor*
**Rasagiline**	**Ras**	**136236-51-6**	$C_{12}H_{13}N$	**171.24**	**Bcl-2 activator**
Revaprazan hydrochloride	*RevHyd*	*178307-42-1*	$C_{22}H_{24}ClFN_4$	*398.9*	*ATPase inhibitor*
**SBI-0206965**	**SBI**	**1884220-36-3**	$C_{21}H_{21}BrN_4O_5$	**489.32**	**ULK1 kinase inhibitor**
Sodium orthovanadate	*SodOrt*	*13721-39-6*	Na_3O_4V	*183.9*	*ATPase inhibitor*
Spautin-1	*Spa*	*1262888-28-7*	$C_{15}H_{11}F_2N_3$	*271.26*	*Bcl-2 inhibitor*
Trichlormethiazide	*Tri*	*133-67-5*	$C_8H_8Cl_3N_3O_4S_2$	*380.66*	*ATPase inhibitor*
Vonoprazan fumarate	*Von*	*1260141-27-2*	$C_{17}H_{16}FN_3O_2S{\cdot}C_4H_4O_4$	*461.46*	*ATPase inhibitor*

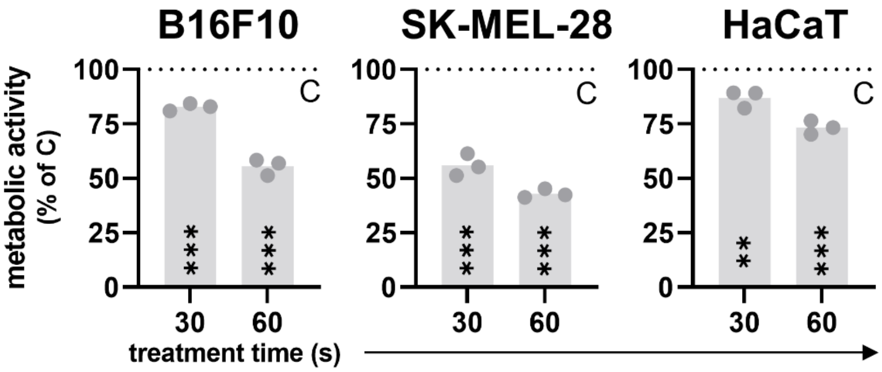

Figure S1. Medical gas plasma technology reduces the metabolic activity of cancer cells with limited effects on healthy keratinocytes. Metabolic activity of B16F10, SK-MEL-28 melanoma cells, and HaCaT keratinocytes 24 h after gas plasma treatment compared to untreated controls (C). Graphs show mean and individual data points. Statistical analysis was performed using one-way analysis of variances (ANOVA) against untreated controls (**$p<0.01$, ***$p<0.001$).

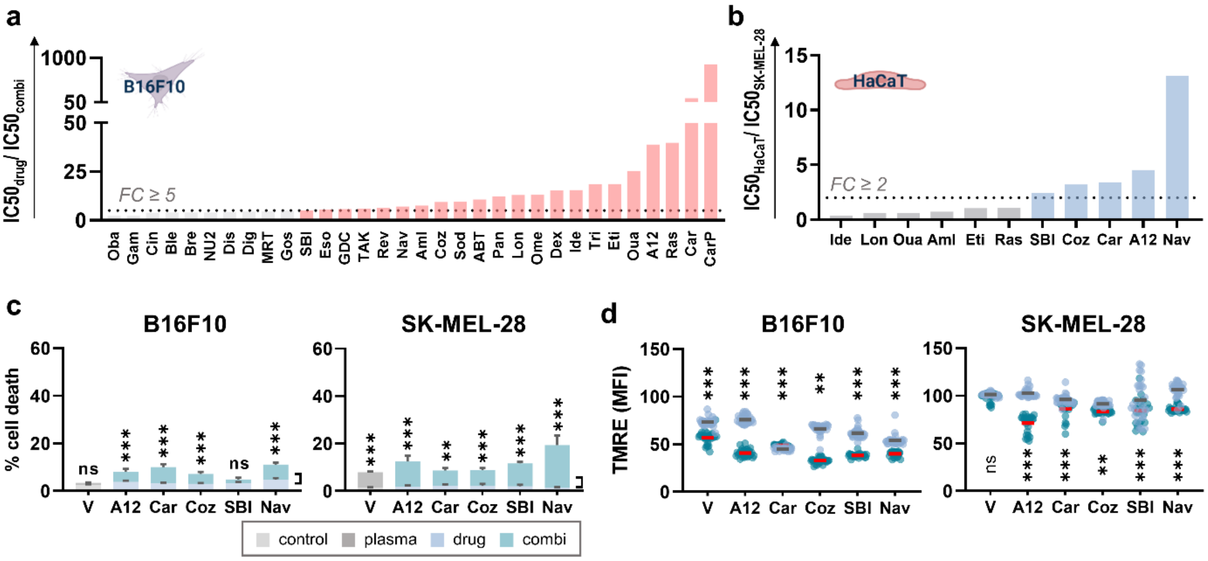

Figure S2. Synergistic effects of mitochondria-targeted drugs and medical gas plasma technology with limited effects on healthy keratinocytes. (**a**) Waterfall plot showing inhibitory concentration (IC) 50 ratios of mono-treatment versus combination regimes in B16F10 murine melanoma cells. Graph shows mean. (**b**) Waterfall plot showing IC50 ratios of combination regimes in HaCaT keratinocytes versus SK-Mel-28 human melanoma cells. Graph shows mean. (**c**) Cell death 6 h after treatment. Bar graphs show mean ± standard error of the mean (SEM). Statistical analysis was performed using two-way analysis of variance (ANOVA) (**p<0.01; ***p<0.001). (**d**) Mitochondrial membrane potential (ΔΨm) 24 h after treatment. Graph shows mean (line) and individual data points. Statistical analysis was performed using two-way analysis of variances (ANOVA) (**p<0.01; ***p<0.001). ns = non-significant. V = vehicle. FC = fold change. TMRE = tetramethylrhodamine. MFI = mean fluorescence intensity.

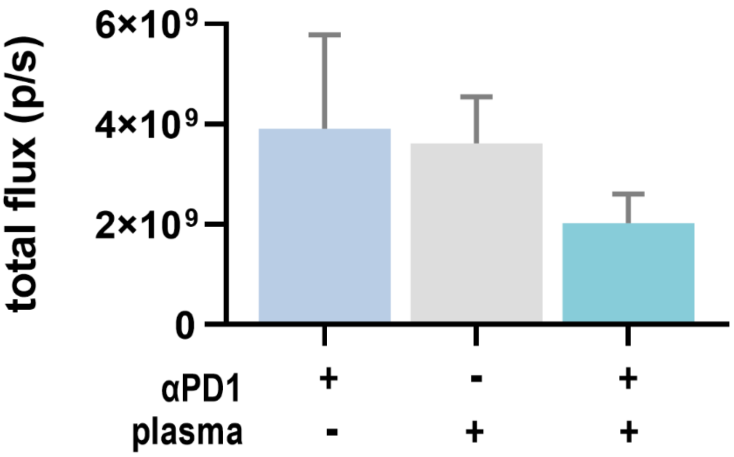

Figure S3. Abscopal effects of combined gas plasma-immune checkpoint blockade. Tumor bioluminescence of the left, non-plasma treated tumor on day 11. Graphs show mean ± standard error of the mean (SEM).
